# Development and evaluation of a loop-mediated isothermal amplification assay for the rapid detection of porcine cytomegalovirus under field conditions

**DOI:** 10.1186/1743-422X-9-321

**Published:** 2012-12-29

**Authors:** Jin-Long Yang, Su-Hui Zhang, Zuo-Hua Liu, Rui Yang, Yong Huang, Ming Wen

**Affiliations:** 1 Chongqing Academy of Animal Science, Chongqing, 402460, China; 2 Animal Science College of Guizhou University, Guiyang, Guizhou, 550025, China

**Keywords:** Porcine cytomegalovirus, Loop-mediated isothermal amplification, Landrace pigs, Field detection

## Abstract

**Background:**

Porcine cytomegalovirus (PCMV) induces silent infection in adult pigs but more frequently causes fatal, generalized infection in newborn piglets. This study aimed to develop a new loop-mediated isothermal amplification (LAMP) method for the sensitive, rapid, and inexpensive detection of PCMV under field conditions.

**Methods:**

Tissue obtained from nine-week-old PCMV-free Landrace pigs or pig samples from postmortem examinations were analyzed. The samples were found to have clinical signs and lesions consistent with inclusion body rhinitis. Six specific primers were designed by targeting the PCMV DNA polymerase (DPOL) DNA. The LAMP reaction was optimized in a water bath. The sensitivity and specificity of LAMP and polymerase chain reaction (PCR) were compared.

**Results:**

PCMV DNA was amplified at 65°C, and the result could be detected as early as 30 min into the reaction. Positive reactions could be visualized by the naked eye as a color change brought on by the addition of SYBR Green. The sensitivity and specificity of LAMP were found to be similar to those of the PCR.

**Conclusions:**

LAMP is a high-throughput technique for the detection of PCMV and has a high specificity, sensitivity and simplicity; these factors make it suitable for detection of PCMV under field conditions.

## Background

Porcine cytomegalovirus (PCMV) is a *β*-herpesvirus that was first described in 1955 [1]. It shares sequence homology with human herpesviruses 6 and 7 and human cytomegalovirus (HCMV) [2-6]. It usually induces silent infection in adult pigs but often a fatal, generalized infection in newborn piglets, with 90% of pigs in the UK being seropositive [3,7]. PCMV has been known to cross the placenta and infect the fetuses in pregnant sows, leading to fetal death or birth of weak piglets [5,6].

PCMV is of particular concern because, similar to its human counterpart, it is characterized by high seroprevalence in the swine population, has diverse clinical manifestations, and establishes latent infection [8,9]. It is one of the major causes of morbidity and mortality in immunocompromised pigs [10]; therefore, diagnosis at an early stage is essential. However, clinical diagnosis of this disease can be difficult, since veterinarians need to consider various underlying diseases and clinical presentations.

Serological detection is one of the most widely used methods for detecting the reactivation of PCMV infection [11-13]. In addition to this technique, nucleic acid-based methods such as polymerase chain reaction (PCR) are increasingly being used for identifying and detecting the pathogens [14,15]. The accuracy and sensitivity of PCR for the detection of specific diseases are higher than other methods [15-17]. PCR can be applied for detecting PCMV infection, and it is considered more useful than other techniques for the prediction of PCMV [18]. Although PCMV can be effectively and accurately detected in a laboratory setting using these procedures, these methods are time-consuming, laborious and require expensive equipment, rendering them unfavorable for wide-scale use or for use under field conditions. In Japan, the PCR method is not widely used in clinical practice for PCMV detection due to the higher costs involved in this technique, as compared to the serological detection method [19]. Therefore, a simple, rapid, sensitive, economical and practical method is required for the detection of PCMV [20].

Loop-mediated isothermal amplification (LAMP) is a DNA amplification method that was recently developed as a low-cost alternative for the detection of certain diseases. LAMP is a rapid and simple method of specific nucleic acid amplification that can be carried out within an hour [21,22]. Only a simple incubator is required for the LAMP technique [21]. At the end of the reaction, the presence or absence of target DNA is visually judged by change in color after the addition of SYBR Green I to the reaction mixture, or by the appearance of a white magnesium pyrophosphate precipitate; thus, differentiating positive from negative results is relatively easy [23,24]. It also allows for a high degree of specificity in the detection of specific pathogens. Since it only requires simple equipment, DNA detection with this technique is cost-effective. This method has previously been applied for the simple and real-time detection of specific pathogens such as enteroviruses [25,26] and *Trypanosoma brucei gambiense*[27].

Currently, LAMP is used in developing countries for the rapid detection of certain pathogens; however, to the best of our knowledge, no study has investigated the application of LAMP for detecting PCMV. Therefore, we aimed to optimize LAMP with the DNA polymerase (DPOL) target sequence from PCMV strain OF-1 for the simple, rapid, cost-effective and sensitive detection of PCMV under field conditions.

## Methods

### DNA preparation

Ten 9-week-old PCMV-free Landrace pigs were purchased from the Experimental Animal Center (Chongqing Medical University, China). All animal care and study protocols were approved by the Animal Studies Committee of Chongqing, China (Certificate Number 2007–0001). Five of these pigs were orally administered a virulent PCMV strain (laboratory-adapted strain, No. 100218; Research Center of Pig Diseases, Chongqing Academy of Animal Science, China), using 0.1 mL of 10^3^ LD_50_ per pig. The remaining five pigs were orally administered an equal volume of water as the control. Three pigs from each group were sacrificed 48 h post-infection, and their spleen and liver were aseptically removed and stored immediately in 1.5 mL labeled snap-cap tubes. Crude DNA was obtained by boiling: approximately 50 mg of tissue was homogenized in 500 μL of 1% sodium dodecyl sulfate (SDS) in 100 mM Tris–HCl (pH 8.0), boiled for 10 min, and centrifuged at 10,000 *g* for 5 min. The supernatant was transferred to a new tube and used immediately [28,29].

### Preparation of standard DNA template

Briefly, PCMV-infected cells were harvested by centrifugation. The pellets were processed using a DNA extraction kit (Tiangen Biotech, Beijing, China) according to the manufacturer’s recommendations. The genomic DNA pellet was resuspended in 50 μL TE buffer (pH 8.0) [30].

The DNA concentration was determined using a spectrophotometer (Bio-Rad Smartspec-3000; Bio-Rad Laboratories, Hercules, CA, USA) according to the manufacturer’s instructions. On the basis of the molecular weight, the standard DNA copy number was calculated using the equations described by Ke et al. [31]. The standard DNA was diluted, divided into aliquots, and stored at −20°C until analysis [28].

### Design of LAMP primers

Six specific LAMP primers were designed to detect PCMV DNA. A specific define (DPOL) within the PCMV gene (Genbank Accession No. AF268041.2) was selected as the target for LAMP. The LAMP primers were designed with the Primer Explorer V4 program (http://primerexplorer.jp). The sequences and locations of the primers are shown in Table 1. 

**Table 1:** Primers used for LAMP

**Primer**	**Type**	**Length**	**Sequence**
F3	Forward outer	18-nt	5’- TATCTGGTTCTGGCGGAC -3’
B3	Backward outer	23-nt	5’- AGCATATTTCTCTTTCTAGTCTC -3’
FIP	Forward inner (F1c + F2)	46-mer(F1c:25-nt, F2:21-nt)	5’- TAGCAGATGCTTCCATATGGTAATT-GGGCAGATATTGTATACAGGA -3’
BIP	Backward inner(B1c + B2)	43-mer (B1c:22-nt, B2:21-nt)	5’- TTCTAAGTTGGCCTACTTGCCC-ATGAAGGATACACGTGAACAC -3’
LF	Loop Forward	23-nt	5’- ACAACTCCTTAACGATCACCGAA -3’
LB	Loop Backward	25-nt	5’-ATCAGGAAGGTGATAAATGATGGAC -3’

### Optimization of the LAMP reaction

A LAMP amplification kit (Eiken Chemical Co. Ltd., Tokyo, Japan) was used for the LAMP reaction. The reaction was prepared according to the manufacturer’s instructions in a total volume of 25 μL. The final primer concentrations used were described by Yang et al. [32]. The reaction time was optimized by incubating the mixture for 10, 20, 30, 40, 50 or 60 min at a pre-determined temperature (65°C). The reaction temperature was optimized by incubating the mixture at 58, 59, 60, 61, 62, 63, 64 or 65°C for a pre-determined time (60 min). The reaction was terminated by heating at 85°C for 2 min. The LAMP products (5 μL) were electrophoresed on 2% agarose gels and stained with ethidium bromide to determine the optimal reaction conditions.

### Visual observation of LAMP products

Amplified DNA could be visualized as white turbidity of the LAMP reaction mixture as a result of magnesium pyrophosphate, which is a by-product of the reaction. The LAMP amplicons in the reaction tube were visually detected after the addition of 1.0 μL of original SYBR Green I (1,000× dilution; Molecular Probes, Sigma-Aldrich Corporation, St. Louis, MO USA). The color of the solution changed from light orange to green in the presence of the LAMP amplicons, whereas the color of the samples without amplicons did not change. The white turbidity in the reaction mixture was also inspected prior to the addition of SYBR Green I. The reaction mixture (5 μL) was loaded and run on 2% agarose gel, stained with ethidium bromide, and visualized by ultraviolet (UV) transillumination. A charge-coupled device camera (TILL Photonics LLC, Martinsreid, Germany) was used to photograph the results [28].

### PCR detection

The target sequences were amplified by the ABI AmpliTaq Gold DNA polymerase system with a 96-well thermal cycler (Veriti FAST 96-well; Bio-Rad Corp., Hercules, CA, USA). The assay was carried out according to the method of Hamel et al. [15], and a 780 bp target sequence was amplified.

### Specificity and sensitivity of LAMP

The specificity of LAMP was tested using standard DNA templates and templates from other beta herpesviruses, namely, human herpesvirus 6 (HHV-6), HHV-7, and HCMV (Strains were purchased from the National Center for Medical Culture Collection, China). The reaction was performed at 65°C for 60 min. PCR was carried out as described for the control assay.

Tenfold serial dilutions (1 × 10^3^ to 1 × 10^–1^ copies/μL) of standard DNA were used to determine the detection limit of the assay, and 1 μL of each dilution was used for the LAMP assay. The reactions were performed at 65°C for 60 min, and the results of this assay were compared with the PCR results.

### Application of LAMP to detect PCMV in animal tissues

To evaluate the optimal conditions for the detection of PCMV via LAMP, total DNA was extracted from the spleen and liver of experimentally infected pigs at 48 h post-infection. Twenty tissue samples obtained by postmortem examinations of the pigs showed the clinical signs and lesions consistent with inclusion body rhinitis; these samples were also analyzed by LAMP and PCR.

## Results

### Optimal conditions for LAMP reaction

**Figure 1:**
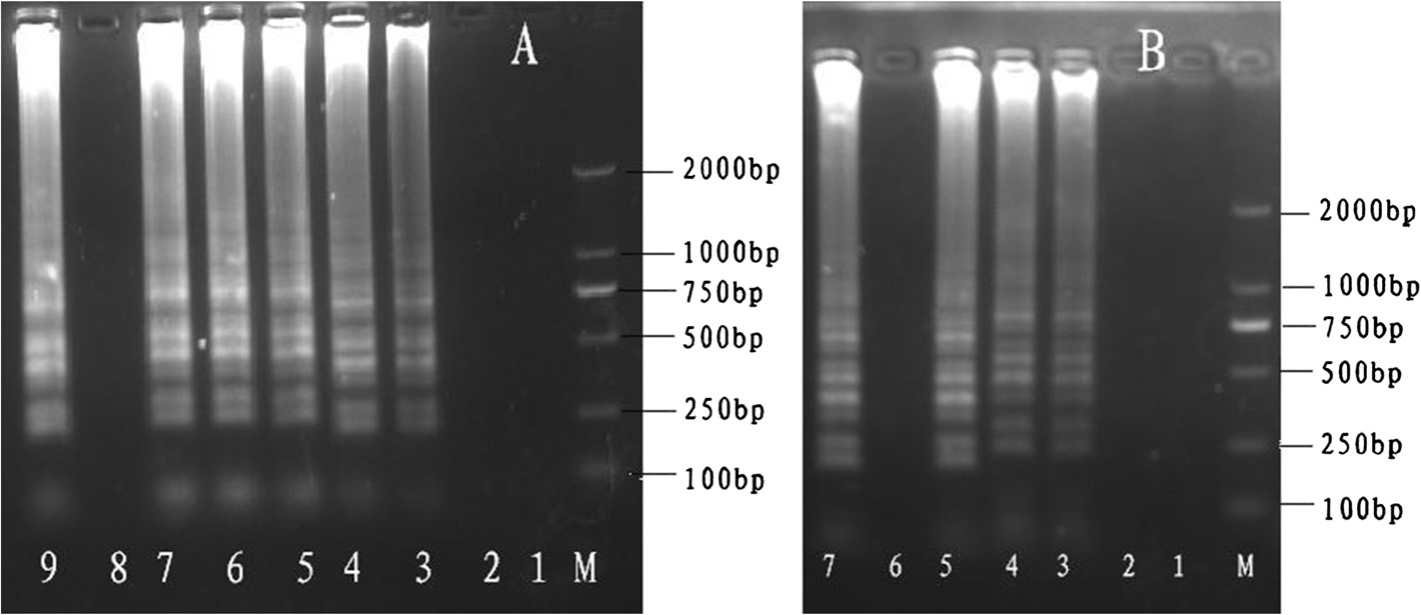
**Determining the optimal temperature and time for LAMP. (A)** Determination of the optimal temperature. Lane M, DL-2000 DNA marker; Lanes 1–9: Reaction for 60 min at 58, 59, 60, 61, 62, 63 and 64°C; negative control; and reaction at 65°C. **(B)** Determination of the optimal time. Lane M, DL-2000 DNA marker; Lanes 1–5 and 7: Reaction for 10, 20, 30, 40, 50 and 60 min (65°C), respectively; Lane 6: –, negative control. All LAMP products were electrophoresed on 2% agarose gels and stained with ethidium bromide.

We determined the optimal temperature and time for the LAMP reaction for the detection of PCMV. Amplicons were formed at 60, 61, 62, 63, 64 and 65°C, but the clearest products were obtained at 63, 64 and 65°C (Figure 1A); the efficiencies of the LAMP reaction at these three temperatures were identical. Meanwhile, LAMP products were detected in the reaction mixture at 65°C within as little as 30 min (Figure 1B). Although well-formed bands could be detected within as early as 30 min, the optimum reaction time at 63–65°C was determined as 40 min to ensure the detection of templates at a lower concentration.

### Specificity and sensitivity of LAMP

**Figure 2:**
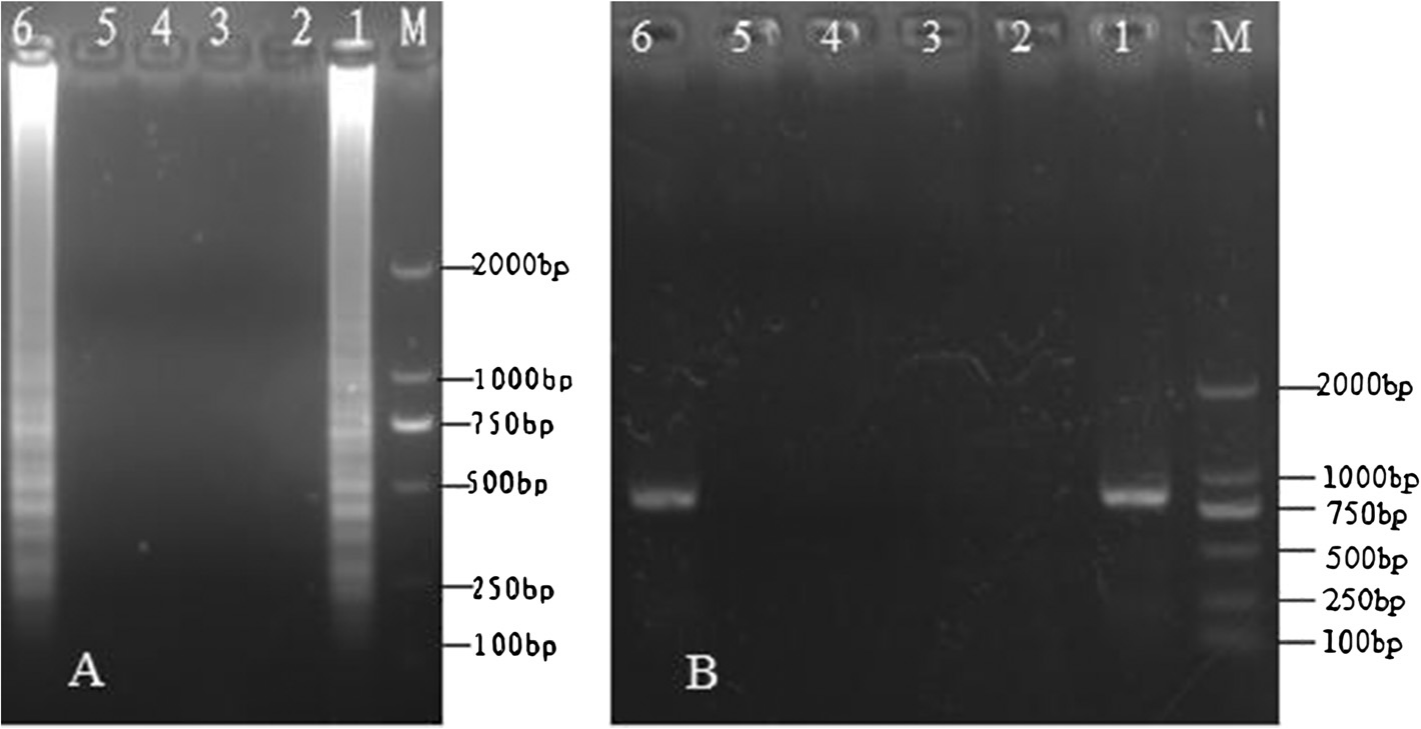
**Specificity of the PCMV-LAMP assay and PCR. (A)** Specificity of LAMP among different species. **(B)** Specificity of PCR among different species. M: DL-2000 DNA ladder marker; 1: +, positive control; 2: –, negative control; 3: human herpesvirus 6 (HHV-6); 4: HHV-7; 5: HCMV; and 6: PCMV. All products were electrophoresed on 2% agarose gels and stained with ethidium bromide.

The specificity of LAMP and PCR was examined for the detection of DNA templates extracted from the four viruses tested (HHV-6, HHV-7, HCMV, and PCMV); however, only PCMV templates tested positive (Figure 2A). The PCR results (Figure 2B) correlated well with those obtained by LAMP. These results indicate that the specificity of the LAMP for the detection of PCMV was similar to that of the PCR assay.

**Figure 3:**
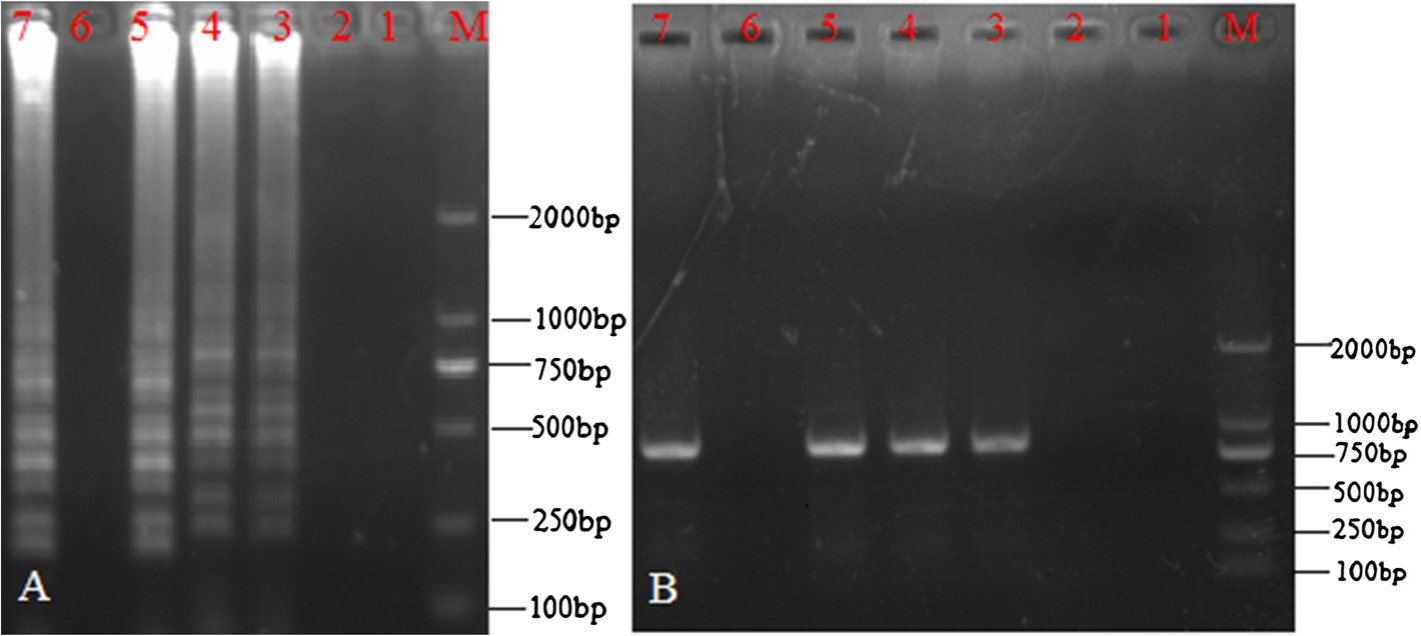
**Sensitivity of the LAMP and PCR. (A)** Sensitivity of LAMP. **(B)** Sensitivity of PCR. M, DL-2000 DNA marker; 1–5, reaction carried out using 10-fold serial dilutions of standard PCMV DNA (1.0 × 10^3^ copies/μL): 1: 1.0 × 10^–1^ copies/μL, 2: 1.0 × 10^0^ copies/μL, 3: 1.0 × 10^1^ copies/μL, 4: 1.0 × 10^2^ copies/μL, 5: 1.0 × 10^3^ copies/μL, respectively; Lane 6: –, negative control, Lane 7: +, positive control. All products were electrophoresed on 2% agarose gels and stained with ethidium bromide.

Furthermore, the detection limits of LAMP and PCR, which were determined using standard DNA templates, were found to be similar (Figure 3). Thus, both these methods showed a similar sensitivity in terms of the detection limit.

### Visual detection of LAMP products

**Figure 4:**
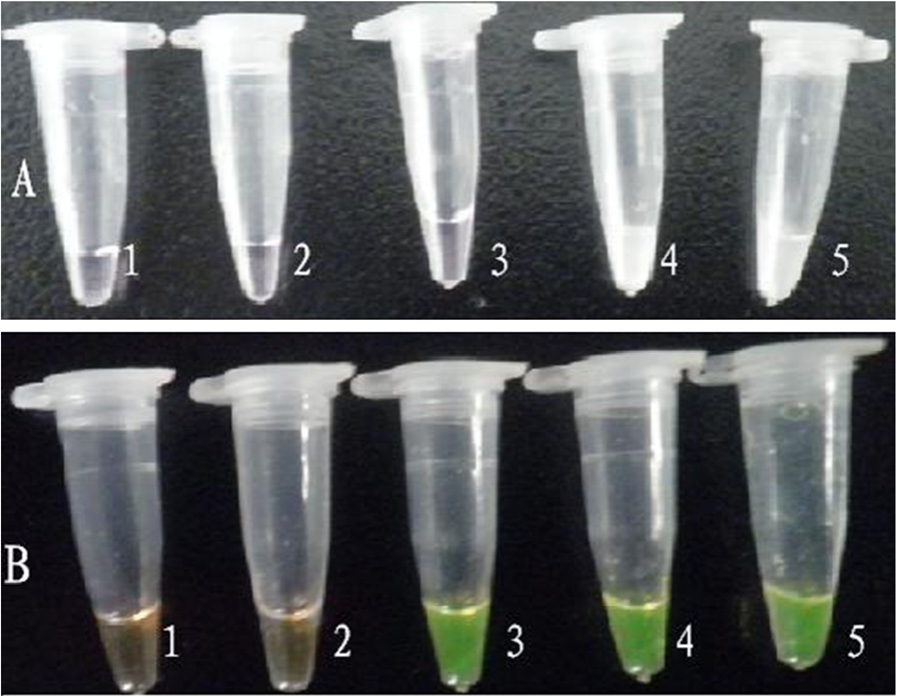
**Detection of LAMP products by observing white turbidity and color of the reaction mixture. (A)** White turbidity of the reaction mixture by magnesium pyrophosphate; **(B)** Green color of the reaction mixture after the addition of SYBR Green I. 1–5, reaction carried out using 10-fold serial dilutions of standard PCMV DNA (1.0 × 10^3^ copies/μL): 1: 1.0 × 10^–1^, 2: 1.0 × 10^0^, 3: 1.0 × 10^1^, 4: 1.0 × 10^2^, and 5: 1.0 × 10^3^ copies/μL.

No white turbidity was observed in the reaction mixtures containing 1.0 × 10^-1^ to 1.0 × 10^1^ copies/μL of standard DNA templates, but white turbidity could be observed in the reaction mixtures containing 1.0 × 10^2^ to 1.0 × 10^3^ copies/μL (Figure 4A). As shown in Figure 4B, the solution color changed from orange to green from 1.0 × 10^1^ to 1.0 × 10^3^ copies/μL of standard DNA template/μL, but not for 1.0 × 10^-1^ to 1.0 × 10^0^ copies/μL. Therefore, the LAMP detection limit was 1.0 × 10^2^ copies/μL with respect to the formation of white turbidity, and 10 copies with SYBR Green I. Thus, the color-change method is 10 times more sensitive than the visual observation method for the detection of amplification products.

### Application of the LAMP assay for PCMV detection

**Figure 5:**
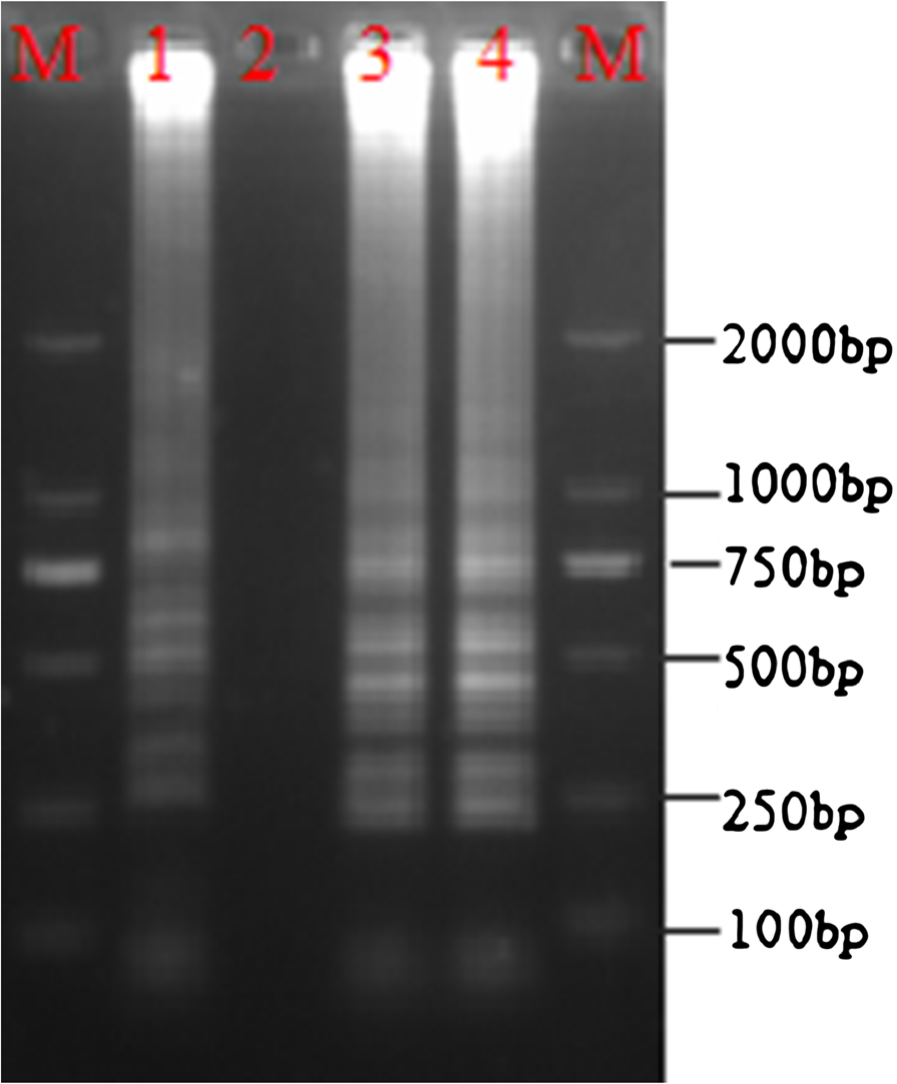
**Detection of PCMV in infected pig tissues by LAMP.** M, DL-2000 DNA marker; 1, positive control; 2, negative control; 3, liver; and 4, spleen.

The application of the LAMP assay was evaluated by analyzing PCMV-infected pig tissues. DNA extracted by the tissue-boiling method showed a typical ladder pattern (Figure 5). The spleen and liver samples tested positive for PCMV. The twenty pig tissue samples obtained by postmortem examination, which exhibited the clinical signs and lesions consistent with inclusion body rhinitis, were analyzed using LAMP and PCR. Of these, 18 tested positive and 2 tested negative in both the PCR and LAMP assays. Results from both LAMP and PCR were consistent (Figure 5).

## Discussion

Since prevention and early detection are the most logical strategies for pathogen control, the most effective method of disease control is routine screening for pathogens [33]. Sensitive and rapid methods are required for the detection of PCMV under field conditions. However, thus far, there is no practical, simple and rapid method for the diagnosis of PCMV under field conditions.

The detection of PCMV DNA is often performed using PCR-based assays, and the majority of these assays are developed in-house. The individual laboratory determines the performance, verification and validation of such assays. As a result, these assays may vary with regard to specimen type, target DNA, nucleic acid extraction method, or detection method. There is a need for a standardized assay to detect PCMV DNA that can be broadly applied in clinical practice and enable the establishment of clinically significant cutoffs [34].

LAMP has successfully been used to diagnose pathogenic infections in humans and animals [35,36]. In our study, no cross-reaction with the other viruses tested was noted in the LAMP assay, similar to the case with PCR. Furthermore, the specificity of LAMP was not affected by non-target genomic DNA in the reaction mixture, which is a highly desirable trait in a diagnostic system [21]. As shown by the results of the present study, the LAMP method was highly sensitive for the detection of PCMV. Consistent with previous reports, LAMP showed the same level of sensitivity as PCR [37,38].

The optimal conditions for PCMV detection by LAMP were determined to be 63–65°C for 40 min. However, the LAMP assay involves fewer steps than the PCR assay, and does not require expensive equipment to attain a high level of precision [39]. The LAMP assay is more rapid than PCR for the detection of animal pathogens, which requires at least 2–3 h for detection [37]. The time required for diagnosis is considered crucial for the diagnosis of pathogenic infections, making LAMP the obvious choice for diagnosing PCMV. In addition, LAMP is ideal for on-site testing, particularly in situations where time is a critical factor, such as when material is subject to quarantine controls.

Furthermore, in the LAMP assay, amplification can be detected as fluorescence by the naked eye, indicating that this assay can be applied in the field. The appearance of color change indicating a positive result occurs after the addition of SYBR Green I; this is a simple and effective method of detecting LAMP amplification products, eliminating the need for gel electrophoresis and ethidium bromide staining [27]. The orange color of the dye changes to green under natural light in the case of a positive reaction [40]. The sensitivity of detection based on the presence of white turbidity was inferior to that based on the color change with SYBR Green I or electrophoresis (Figures 3 and 4); ten-fold more copies of template DNA are required so that a positive reaction in terms of white turbidity can be visually detected. Quantitative detection is difficult in the LAMP assay, but inspection with the naked eye is simple and rapid. LAMP can potentially be used under field conditions even by non-specialists (for example, to carry out surveillance at ports of entry or in the nursery industry) and in small or regional laboratories where nucleic acid-based testing is not currently performed and equipment is limited [41]. No expensive equipment is necessary to obtain a high level of precision equivalent to or greater than that obtained with other PCR techniques. Therefore, this method of detection may facilitate the application of LAMP, especially in the field, where the availability of equipment and expertise may be limited.

A previous study has reported that earlier detection of infection results in earlier treatment and consequently, earlier recovery [37]. Our final goal was to establish a simple and rapid diagnostic method for the specific detection of PCMV under field conditions. To perform LAMP under field conditions, it is crucial to have a suitable nucleic acid extraction technique that requires minimal equipment and can produce sufficient DNA within a short time. The only equipment required in the LAMP assay is a water bath; this is essential for both DNA preparation and nucleic acid amplification. Thus, the LAMP assay can be adopted in most situations where a rapid diagnosis is required, without the need for complicated equipment and technical training. Thus, LAMP is a more rapid method of detection than PCR, even when the PCMV titers are very low. This is due to the high sensitivity of LAMP and its detection limit of approximately 10 copies. We recommend that this technique be applied routinely for the early detection of PCMV, so that adequate countermeasures can be adopted before infections become epizootic [31]. Nevertheless, it must be clarified that the viral copy numbers in this study are not accurate, because we did not use a purified recombinant plasmid containing the target gene for these assays. Further studies with a purified recombinant plasmid are required to improve this assay technique.

In summary, the LAMP protocol described here is a new, inexpensive, and rapid method with high sensitivity and specificity for the detection of PCMV. No complicated technical operations, experimental conditions, or special equipment is required for this technique; only a simple water bath is necessary. Therefore, LAMP is an advantageous diagnostic tool for the specific detection of PCMV infection in animals under laboratory and field conditions.

## Competing interests

The authors declare that they have no competing interests.

## Authors' contributions

JLY, ZHL, SHZ and RY carried out most of the experiments and wrote the manuscript. MW critically revised the manuscript and the experiment design. All of the authors read and approved the final version of the manuscript.
